# Enhanced Butanol Production by *Clostridium acetobutylicum* NCIMB 13357 Grown on Date Fruit as Carbon Source in P2 Medium

**DOI:** 10.1155/2014/395754

**Published:** 2014-01-06

**Authors:** Emran I. Khamaiseh, Aidil Abdul Hamid, Peyman Abdeshahian, Wan Mohtar Wan Yusoff, Mohd Sahaid Kalil

**Affiliations:** ^1^School of Biosciences and Biotechnology, Faculty of Science and Technology, Universiti Kebangsaan Malaysia, 43600 Bangi, Selangor, Malaysia; ^2^Department of Chemical and Process Engineering, Faculty of Engineering and Built Environment, Universiti Kebangsaan Malaysia, 43600 Bangi, Selangor, Malaysia

## Abstract

The production of biobutanol was studied by the cultivation of *Clostridium acetobutylicum* NCIMB 13557 in P2 medium including date fruit as the sole substrate. The effect of P2 medium and the effect of different concentrations of date fruit ranging from 10 to 100 g/L on biobutanol production were investigated. Anaerobic batch culture was carried out at 35°C incubation temperature and pH 7.0 ± 0.2 for 72 h. Experimental results showed that the lowest yield of biobutanol and acetone-butanol-ethanol (ABE) was 0.32 and 0.35 gram per gram of carbohydrate consumed (g/g), respectively, when an initial date fruit concentration of 10 g/L was utilized. At this fruit date concentration a biobutanol production value of 1.56 g/L was obtained. On the other hand, the maximum yield of biobutanol (0.48 g/g) and ABE (0.63 g/g) was produced at 50 g/L date fruit concentration with a biobutanol production value as high as 11 g/L. However, when a higher initial date fruit concentration was used, biobutanol and ABE production decreased to reach the yield of 0.22 g/g and 0.35 g/g, respectively, where 100 g/L date fruit was used. Similar results also revealed that 10.03 g/L biobutanol was produced using 100 g/L date fruit.

## 1. Introduction

The worldwide interest has been shown for finding sustainable and low-cost energy sources with reducing the global warming. Therefore, nowadays a number of attempts have been made for using biofuels as petroleum-derived transportation fuels [1]. Among the varieties of biofuels which are currently available, bioethanol as a partial replacement gasoline has gained the attention of commercial sectors during the last few years. In this regard, much progress has been made in terms of improved strain development, fermentation processes for cellulosic biomass, and separation techniques [2, 3]. Biobutanol is a superb biofuel with many advantages over bioethanol, such as higher energy content, higher blending rate with gasoline without engine modification, convenient distribution using current pipeline infrastructure, and better auto emission performance [4].

Butanol is commonly produced in acetone-butanol-ethanol (ABE) fermentation process. ABE fermentation process is one of the main biochemical processes of biofuel production which has found industrial application [5]. Biobutanol is produced by microorganisms in ABE fermentation process using sustainable carbohydrate sources such as lignocellulosic biomass. It has been shown that *Clostridium* species are capable of producing biobutanol [6]. ABE fermentation process includes two phases. The first phase is known as the acidogenic phase. During this phase, the acid formation pathways are activated in which carbohydrate substrates particularly glucose are fermented to organic acids. Acetate, butyrate, hydrogen, and carbon dioxide are the major products of this phase. This acidogenic phase usually occurs during the exponential growth phase of *Clostridium* species. The second phase is the solventogenic phase in which acids reassimilation occurs. The products obtained in this phase are mainly acetone, butanol, and ethanol [7].

A large quantity of ABE production costs are related to substrate costs. Hence, the utilization of a low-cost renewable carbohydrate source can decrease biobutanol production costs. The date palm (*Phoenix dactylifera* L.) has been found to be an important bioenergy production source since it contains a high percentage of carbohydrate with a total sugar percentage of 70%. Fruit dates also consist of fat (0.2–0.5%), salts and minerals (15%), protein (2.3–5.6%), and vitamins and a high percentage of dietary fiber (6.4–11.5%). The flesh of dates contains 0.2–0.5% oil, whereas the seed contains 7.7–9.7% oil [6, 8]. Although a number of carbohydrate-based bioenergy sources have been utilized for biobutanol production [9, 10], much less work has been performed to use palm dates for biobutanol production in ABE fermentation process [6]. In the previous study, the optimization of process parameters including initial pH of culture and incubation temperature for biobutanol product using fruit date was performed [11]. The current study aimed at enhancing biobutanol production by *C. acetobutylicum* NCIMB 13357 from date palm in ABE fermentation. The effect of the defined medium, namely P2 medium, on butanol production was also investigated.

## 2. Materials and Methods

### 2.1. Microorganism

*C. acetobutylicum* NCIMB 13357 was provided by the Biotechnology Laboratory, Chemical and Process Engineering Department of UKM. Fresh inoculum suspension was prepared by maintaining *C. acetobutylicum* NCIMB 13357 in reinforced clostridium medium (RCM) [14]. Prepared RCM was then incubated at 35°C for 24 hours under anaerobic conditions and directly used in the experiments. The inoculum size of 10% was used to inoculate the fermentation medium.

### 2.2. Substrate and Medium Preparation

Dried date fruit of 100 g was blended and mixed in distilled water to make the final volume of 1000 mL. It was then filtered to remove solid particles and mixed with synthetic P2 medium in known proportion to prepare a final fermentation medium containing varied date fruit concentrations ranging from 10 to 100 g/L. Synthetic P2 medium had the following composition (in g/L): yeast extract, 1; KH_2_Po_4_, 0.5; K_2_HPO_4_, 0.5; para aminobenzoic acid, 0.001; thiamin, 0.001; biotin, 1 × 10^−5^; MgSo_4_·7H_2_O, 0.2; MnSo_4_·7H_2_O, 0.0l; Fe_2_So_4_·7H_2_O, 0.01; NaCl, 0.01; and ammonium acetate, 2.2 [15].

### 2.3. Biobutanol Production

Fermentation process was carried out by transferring 150 mL of P2 medium including different concentrations of date fruit (10 to 100 g/L) to a 250 mL Duran Schott bottle. The initial pH of medium was adjusted at pH 7.0 ± 0.2 by an addition of 0.2 M NaOH and 0.1 M HCl. It was then autoclaved at 121°C for 15 minutes and allowed to cool to room temperature. Anaerobic condition was achieved by sparging oxygen-free nitrogen gas into medium. *C. acetobutylicum* NCIMB13357 with an inoculation size of 10% was cultivated in P2 medium at the temperature of 35°C for 72 hours. Culture broth was then harvested to perform analytic methods.

### 2.4. Analytic Methods

Anthrone method [16] was used to determine the amount of the total carbohydrate consumed during the fermentation process. Solvents and acids concentrations were determined by gas chromatograph with capillary column (Equity-1 Supelco) as previously described [17]. The yield of products was calculated as the total ABE or butanol produced divided by the total carbohydrate utilized. The yield of products was expressed as gram/gram. The productivity of butanol and ABE was calculated as the total ABE and butanol (gram/Liter) divided by fermentation time (hour) which was expressed as g/L/h.

## 3. Results and Discussion

### 3.1. Biobutanol Production

As previously mentioned, the production of *clostridial* solvent is a biphasic process. At the first phase (the acidogenic phase) acids forming pathways are activated in which acetate, butyrate, hydrogen, and carbon dioxide are produced as major products. This acidogenic phase usually occurs during the exponential growth phase [18, 19]. The second phase is the solventogenic phase during which acids are reassimilated and used in the production of acetone, butanol, and ethanol. During ABE fermentation processes, *Clostridium acetobutylicum* can utilize the sugar (carbohydrate source) in the medium and convert it to acetone, ethanol, and butanol.

**Figure 1:**
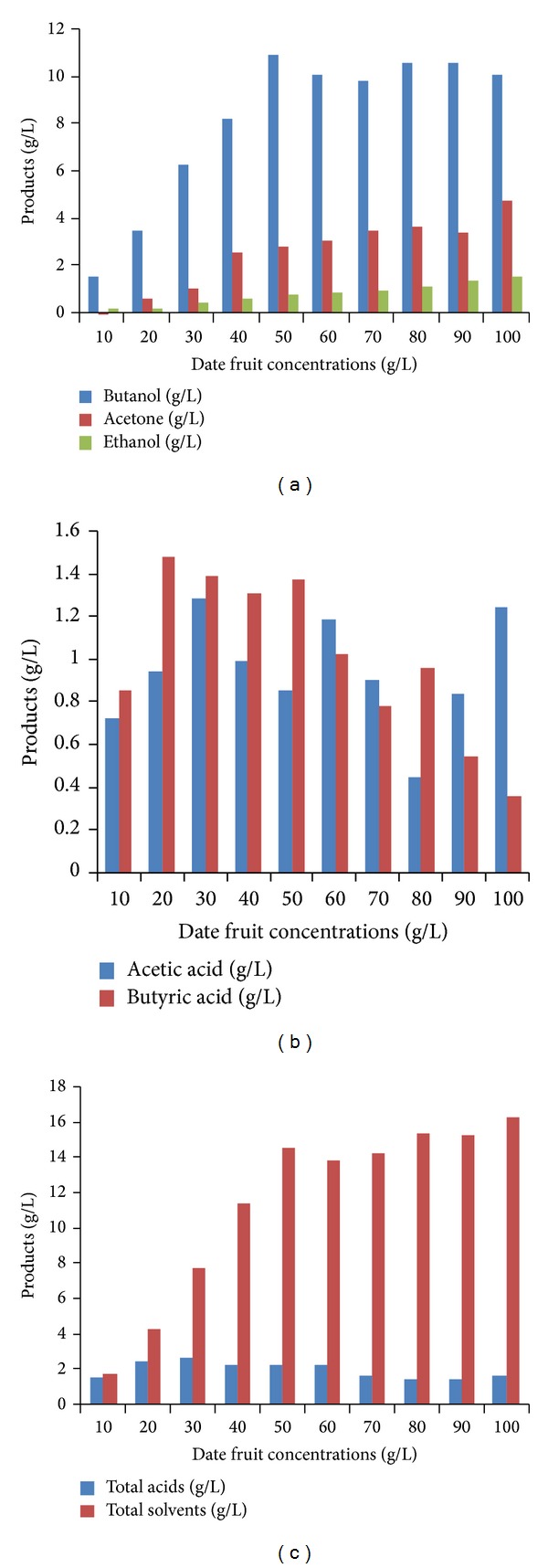
ABE fermentation by *C. acetobutylicum* NCIMB 13557: (a) production of butanol, ethanol, and acetone, (b) production of acetic acid and butyric acid, and (c) total acids and total solvents production.

The production of butanol and other solvents by *C. acetobutylicum* NCIMB13357 in this study is shown in Figure 1(a).  Figure 1(b) illustrates acetic acid and butyric acid production. Total ABE and total acids production are depicted in Figure 1(c). As shown in Figure 1(a), the highest level of butanol (11 g/L) was obtained at 50 g/L date fruit, while fermentation culture exhibited the lowest amount of butanol (1.56 g/L) at l0 g/L of date fruit with the production of ethanol and acetone values of 0.13 g/L and 0.02 g/L, respectively. The low amount of fermentation products at a relatively low initial date fruit concentration can be attributed to the low amount of carbon source which represents mass action [20]. Obviously, the production of butanol increased drastically with increasing date fruit concentration from 10 g/L to 50 g/L indicating that the production of butanol in anaerobic fermentation was accompanied by the utilization of the carbohydrate source (date fruit) mixed in P2 medium as supplement media. Hence, the initial carbohydrate concentration performed an important role in the amount of butanol production during the fermentation process. However, an increase in carbohydrate source at higher concentrations than 50 g/L date fruit had no considerable increase in butanol production which was possibly because of keeping constant microbial metabolism for butanol production. As can be found, increased date fruit concentrations from 10 to 100 g/L concurrently caused a slow rise in ethanol and acetone production to obtain the highest concentration ethanol and acetone with values as high as 1.52 g/L and 4.74 g/L, respectively, using 100 g/L date fruit. The lower butanol production at higher level of date fruit can be explained by the fact that the carbon flux at high carbohydrate concentrations causes microbial metabolism to be more directed to the production of reduced by-products such as ethanol and organic acids [21]. Experimental results also revealed that the maximum concentrations of butyric acid (1.47 g/L) and acetic acid (1.28 g/L) were produced at 20 g/L and 30 g/L of date fruit concentration, respectively (Figure 1(b)). As shown, the values of 0.86 g/L and 1.38 g/L were measured for the acetic acid and butyric acid, respectively, when 50 g/L of date fruit was used. As can be seen from Figure 1(c), the highest concentration of total solvent production (16.29 g/L) was obtained when 100 g/L fruit date was utilized. Formanek et al. [22] reported that the amounts of butanol, acetone, and ethanol produced by *C. beijerinckii* BA101 were 18.6 g/L, 8.6 g/L, and 0.3 g/L, respectively, using semidefined P2 medium containing 6% maltodextrin or glucose. However, in the same study fulfilled by Formanek et al. [22] 9.2 g/L butanol, 4.4 g/L acetone, and 0.9 g/L ethanol were produced when *C. beijerinckii* 8052 was cultivated.

As can be seen, at 30 g/L of date fruit the amount of butanol produced was 6.26 g/L and a total solvent was 7.72 g/L (Figure 1). Similar to this study, Al-Shorgani et al. [23] found that the amount of 5.36 g/1 biobutanol and 7.57 g/L total solvents was produced by cultivation of *C. acetobutylicum* in P2 medium containing 30 g/L of glucose. As shown in Table 1, the total sugar consumed by *C. acetobutylicum* had a progressive pattern from 10 to 100 g/L date fruit concentration, while the maximum butanol yield reached up to 50 g/L date fruit concentration and a higher addition of carbohydrate source had no favorable effect on enhanced butanol production implying that 50 g/L date fruit provided an optimum sugar concentration for the highest butanol production. This finding could be attributed to the changes in microbial metabolism of carbon source to the reduced efficiency of metabolic activity known as overflow metabolism [24]. The increasing sugar consumption despite no increase in biobutanol production higher than 50 g/L date fruit could be related to sugar consumed for the production of acetone and ethanol (Figure 1).

### 3.2. Effect of Medium

Fermentation medium has been shown to perform significant effect on fermentation process and product formation. Regarding enhancing butanol production by *C. acetobutylicum* NCIMB 13357 using date fruit as carbohydrate source, Khamaiseh et al. [13] found that the maximum biobutanol production by *Clostridium acetobutylicum *was 5.31 g/L using modified medium containing 40 g/L date fruit (Table 2). They also observed that total solvent and total acids were 8.5 g/L and 5.0 g/L, respectively, with 16.3 g/L sugar consumed (Table 3). In a similar study carried out by Khamaiseh et al. [11] *C. acetobutylicum *was cultivated in reinforced *clostridial* medium (RCM) containing date fruit as a carbohydrate source. They found that the maximum concentration of butanol produced by *C. acetobutylicum* was 4.4 g/L using 40 g/L date fruit (Tables 2-3). In comparison to previously mentioned studies, this research study has shown a considerable increase in butanol and total solvent production *by C. acetobutylicum* using P2 medium indicating the high ability of *C. acetobutylicum* for butanol production by consuming a relatively low concentration of date fruit as carbohydrate source.

On the other hand, as shown in Table 3, the productivity of butanol and ABE increased using date fruit mixed in P2 medium compared to that when modified date fruit medium and date fruit mixed in RCM were used indicating the enhancement of product formation by *C. acetobutylicum* when P2 medium was employed.

The results obtained from this study also showed that the yield of biobutanol and ABE increased compared to products yield measured in previous studies using modified date fruit medium and RCM broth (Table 3). The glucose has been observed to be the most favorable substrate for butanol production by *Clostridium acetobutylicum*. Moreover, it has been shown that yeast extract and mineral supplements such as K_2_HPO_4_, NaCl, and MgSO_4_ have significant effect on the increment of biobutanol production by *Clostridium* species [10, 25]. Consequently, P2 medium had favorable supplements for the growth of *C. acetobutylicum* to produce more butanol and solvents and to cause higher productivity of butanol and ABE. From the results presented it is deduced that butanol production is dependent on both the type of medium and the concentration of substrates used.

### 3.3. Effect of Incubation Time

**Figure 2:**
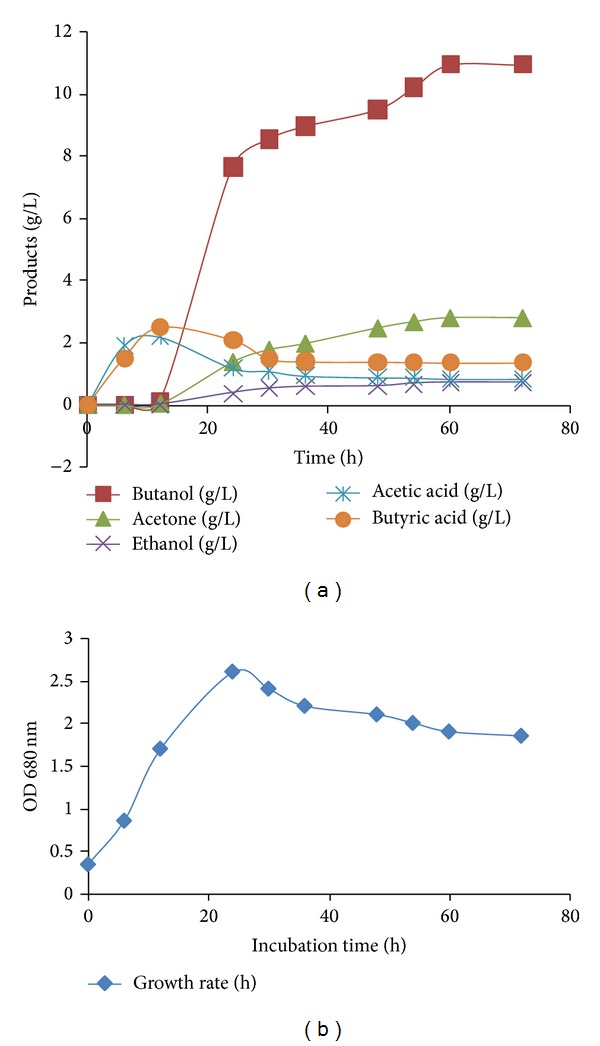
Production of ABE from date fruit by *C. acetobutylicum* NCIMB13357: (a) products at various fermentation times and (b) fermentation time and microbial growth curve based on optical density values (OD) at 680 nm.

In order to study the effect of fermentation time on the production of butanol, solvents and acids by *C. acetobutylicum, *batch culture fermentation was run using 50 g/L date fruit in P2 medium at 35°C for 72 h. Fermentation culture was collected every 6 h to carry out analytical assay. Tables 4-5 represent product formation during 72 h fermentation. As can be seen, the maximum concentration of total acids produced during fermentation was 4.7 g/L after 12 h fermentation process. Furthermore, the maximum concentrations of biobutanol (11 g/L), ethanol (0.74 g/L), and acetone (2.83 g/L) productions were obtained after 60 h fermentation with the total solvents concentration of 14.51 g/L. As is evident, the product formation was kept constant to the end of the fermentation process. Similar pattern was observed for the yield of butanol and ABE (solvents) (Table 4). As shown, the maximum yield of butanol and ABE was 0.44 and 0.58 g/g, respectively, after 60 h fermentation process. Table 4 also shows that the highest productivity of butanol 0.32 (g/L/h) and ABE (0.39 g/L/h) was measured after 24 h fermentation process.  Figure 2 depicts the fermentation time process in relation to product formation studied by *C. acetobutylicum* using P2 medium during 72 h fermentation process. As can be found, the highest amounts of acetic acid and butyric acid were produced during 20 h fermentation time (Figure 2(a)), which is consistent with exponential growth rate (Figure 2(b)), suggesting the high ability of *C. acetobutylicum* to consume the carbohydrate source of date fruit for acid formation using P2 medium. As is evident from Figure 2, the reassimilation of acids remarkably increased after 20 h fermentation time up to maximum production at 60 h implying that microbial pathway of solventogenic phase intensified at stationary phase of growth rate.

## 4. Conclusions

This study showed fermentative butanol production by *C. acetobutylicum *NCIMB 13557 from date fruit using P2 medium as a nutrient supplement. The maximum butanol produced was 11 g/L using 50 g/L date fruit mixed in P2 medium. At this fruit date concentration the highest yield and productivity of butanol were 0.48 g/g and 0.16 g/L/h, respectively. The results of this study could be applied to the development of the butanol production process to obtain more amounts of butanol.

## Figures and Tables

**Table 1:** Effect of substrate concentrations on yield and productivity of butanol and ABE.

DF concentration (g/L)	TC consumption (g/L)	Yield of solvents (g/g)	Yield of butanol (g/g)	Productivity of solvents (g/L/h)	Biobutanol productivity (g/L/h)
10	4.965	0.35	0.32	0.025	0.023
20	9.7	0.45	0.36	0.062	0.05
30	15	0.53	0.42	0.11	0.09
40	18.9	0.61	0.44	0.17	0.12
50	23.2	0.63	0.48	0.21	0.16
60	25.4	0.55	0.4	0. 2	0.15
70	29.6	0.49	0.34	0.2	0.14
80	35.4	0.44	0.3	0.22	0.152
90	40.2	0.38	0.27	0.22	0.15
100	47.6	0.35	0.22	0.24	0.15

DF: date fruit concentration; TC: total carbohydrate; RCM: reinforced clostridial medium.

**Table 2:** A comparison of butanol, acetone, ethanol, acetic acid, and butyric acid production from date fruit with different modified media in batch culture using *C. acetobutylicum* NCIMB 13357.

Medium	Date fruit concentration (g/L)	Butanol (g/L)	Acetone (g/L)	Ethanol (g/L)	Acetic acid (g/L)	Butyric acid (g/L)	References
Date fruit	30	3.1	1.1	0.1	0.5	0.6	Khamaiseh et al. [12]
RCM-DF	40	4.4	1.33	0.478	0.46	1.32	Khamaiseh et al. [11]
P2 medium	50	11.0	2.84	0.75	0.86	1.37	This study
Modified-DF	40	5.31	1.95	1.17	2.0	3.1	Khamaiseh et al. [13]

DF: date fruit; RCM: reinforced clostridial medium.

**Table 3:** A comparison of ABE and acids production from date fruit with different modified media in batch culture using *C*.  *acetobutylicum* NCIMB 13357.

Medium	Date fruit concentration (g/L)	TC consumed (g/L)	Biobutanol (g/L)	Total solvents (g/L)	Total acids (g/L)	ABE productivity (g/L/h)	Butanol productivity (g/L/h)	ABE yield (g/g)	Butanol yield (g/g)	References
DF	30	13.8	3.1	4.3	1.1	0.062	0.045	0.45	0.32	Khamaiseh et al. [12]
RCM-DF	40	15.07	4.4 g/L	6.2	1.8	0.09	0.07	0.41	0.3	Khamaiseh et al. [11]
P2 medium	50	23.2	11 g/L	14.5	2.2	0.21	0.16	0.63	0.48	This study
Modified-DF	40	16.3	5.31	8.5	5	0.12	0.08	0.51	0.33	Khamaiseh et al. [13]

DF: date fruit; RCM: reinforced clostridial medium; TC: total carbohydrate.

**Table 4:** ABE fermentation by *C. acetobutylicum* NCIMB 13357 using 50 g/L date fruit in P2 medium.

Time (h)	Sugar consumed (g/L)	Productivity of butanol (g/L/h)	Productivity of ABE (g/L/h)	Yield of butanol (g/g)	Yield of ABE (g/g)
0	0	0	0	0	0
6	9.9	0.001	0.005	0.001	0.003
12	14.7	0.007	0.013	0.006	0.010
24	19.9	0.320	0.393	0.384	0.474
30	22.56	0.284	0.362	0.378	0.482
36	24.0	0.249	0.321	0.373	0.481
48	24.6	0.197	0.262	0.386	0.513
54	24.9	0.188	0.252	0.409	0.546
60	25.1	0.182	0.241	0.44	0.58
72	25.1	0.151	0.201	0.44	0.58

**Table 5:** Product formation by *C. acetobutylicum* NCIMB 13357 in ABE fermentation process using 50 g/L date fruit in P2 medium.

Time	Sugar consumed (g/L)	Butanol (g/L)	Acetone (g/L)	Acetic acid (g/L)	Butyric acid (g/L)	Ethanol (g/L)	Total acid (g/L)	Total solvents (g/L)
0	0	0.01	0.01	0.01	0.01	0.01	0.02	0.03
6	9.9	0.01	0.01	1.9	1.5	0.01	3.4	0.03
12	14.7	0.09	0.05	2.2	2.5	0.02	4.7	0.16
24	19.9	7.64	1.4	1.2	2.1	0.4	3.3	9.44
30	22.56	8.54	1.8	1.1	1.5	0.54	2.6	10.88
36	24	8.96	2.0	0.95	1.4	0.6	2.35	11.56
48	24.6	9.5	2.5	0.9	1.39	0.62	2.29	12.62
54	24.9	10.2	2.7	0.89	1.38	0.71	2.27	13.61
60	25.1	11.0	2.83	0.85	1.36	0.74	2.21	14.51
72	25.1	11.0	2.83	0.85	1.36	0.74	2.21	14.51
